# Increased Accuracy in the Automated Interpretation of Large EPMA Data Sets by the Use of an Expert System

**DOI:** 10.6028/jres.093.037

**Published:** 1988-06-01

**Authors:** K. Janssens, W. Van Borm, P. Van Espen

**Affiliations:** Department of Chemistry, University of Antwerp (UIA), Universiteitsplein 1, B-2610 Wilrijk/Antwerp, Belgium

## 1. Introduction

Characterization of particulate material is one of the major applications of Electron Probe Micro Analysis (EPMA). This involves the collection of an energy dispersive x-ray spectrum for each particle to determine its chemical composition. Since for each aerosol sample typically 1000 particles are measured, very large data sets are obtained. Because of limitations in computer time and mass storage capacity, these spectra are not stored but are processed on-line, i.e., they are converted into tables of peak energies and intensities, permitting the characterization of 1000 particles in 4 hours of instrument time without operator intervention. The off-line data processing consists of the interpretation of the peak table associated with each particle in terms of their chemical constituents. By using the Kα or Lα lines of each element, a particle vs elemental x-ray intensity matrix is built which is issued as input for multivariate classification techniques.

Since almost all detailed spectral information is lost in the initial data reduction process, qualitative interpretation by a conventional computer program produces erroneous results when peak overlap occurs. However, as human interpreters can obtain better results based on the same limited information, it was decided to capture the additional interpretation knowledge used by the chemist into an expert system, implemented in the OPS5-language [1], Before the expert system starts an interpretation session, the peak tables generated during the on-line data reduction phase (see table 1a) are converted into a representation which is more suitable for the expert system. For each peak, a library of principal x-ray lines is searched. Since each peak can be associated with several types of x-ray lines of different elements (e.g., a peak at 1.479 keV corresponds to Al-K or Br-Lα), a (sparse) matrix of possible identifications is obtained (see table 1b). These matrices are read directly by the expert system.

## 2. The Expert System

**Figure 1:**
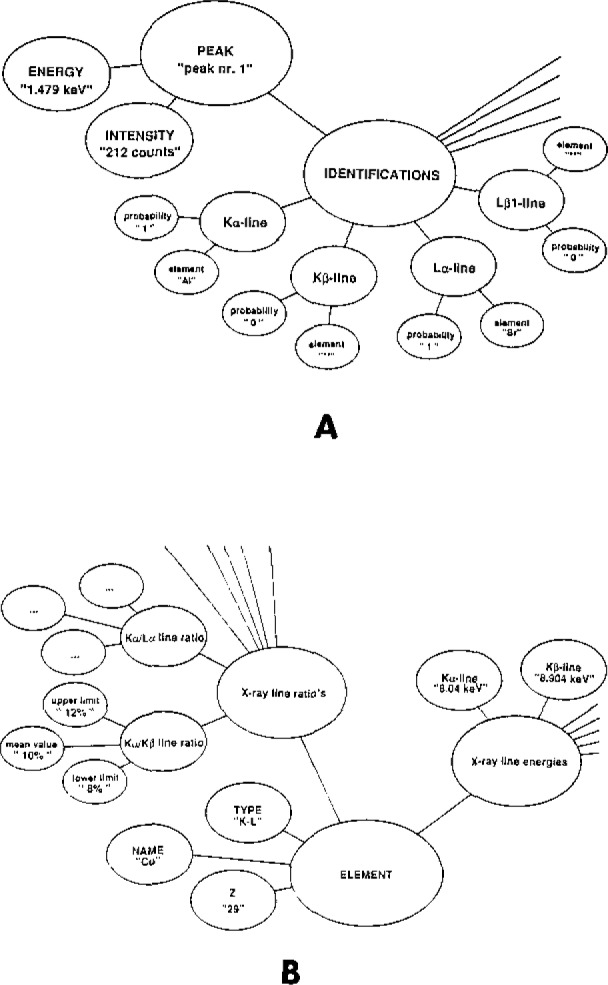
Structure of the PEAK and ELEMENT-type of data objects used by the expert system to store peak- and element-specific information.

Inside the expert system’s data base, the data present in each row of an identification matrix are stored into an OPS5 working memory element (WME) of type “PEAK.” WME’s are complex data structures having several distinct fields. The structure of a peak-WME is represented in figure 1a. As in the identification matrices, each peak can be associated with seven elements. A probability value (e.g., p_Ka_, p_Kb_, …) corresponds with every association. X-ray data pertaining specifically to a chemical element is stored into WME’s of type “ELEMENT” (see fig. 1b).

**Figure 2:**
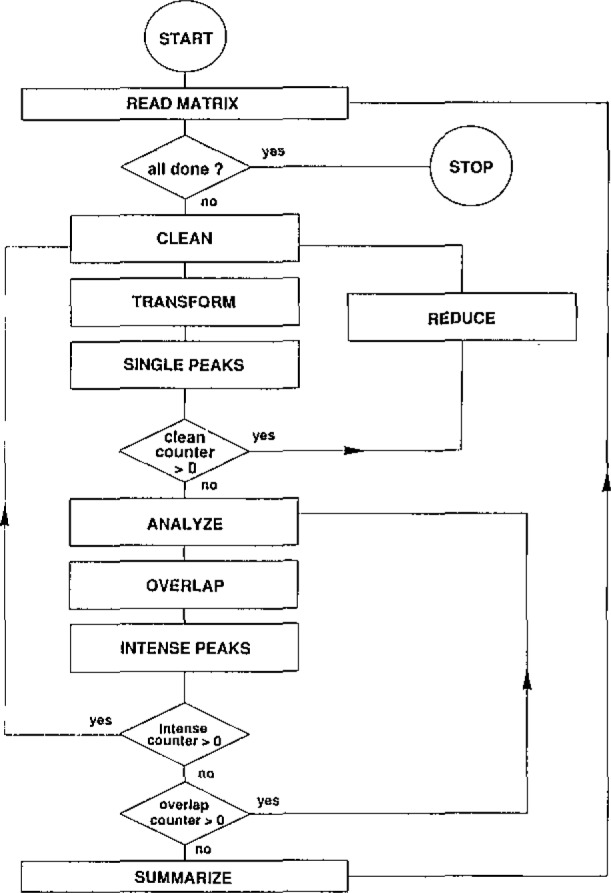
Schematic overview of the interaction between the rule modules present in the expert system’s knowledge base.

Schematically, the functioning of the expert system is represented in figure 2. The systems production rules are organized in several modules (e.g., CLEAN, ANALYZE, OVERLAP, …) each dealing with a particular phase of the interpretation. Interaction between the modules is handled by meta-rules. Table 2a lists a meta-rule and its OPS5-equivalent. At present, the knowledge base contains about 80 chemical knowledge rules. Table 2b lists a rule from the CLEAN module. By using these rules, the system decreases/increases the probability values of a peak as more evidence is found that the associated chemical element is absent/present. The functioning of the expert system is described in more detail elsewhere [2].

## 3. Results and Discussion

The performance of the expert system (method A) was evaluated by comparing the expert system results with those obtained by manual interpretation (method B) and by a conventional FORTRAN interpretation program (method C), using aerosol samples collected in a suburban area [3]. The conventional program operates by summing all peak tables of a data set, yielding a summary spectrum. A set of windows is constructed in which the peak intensities are accumulated while the window positions and widths are continuously adjusted during the summing process. After the summation, each window is associated with a chemical element by comparing its mean energy with energies of principal x-ray line energies after which the elements associated with each individual particle are determined. Thus, a particle vs elemental x-ray intensity matrix is obtained.

**Figure 3:**
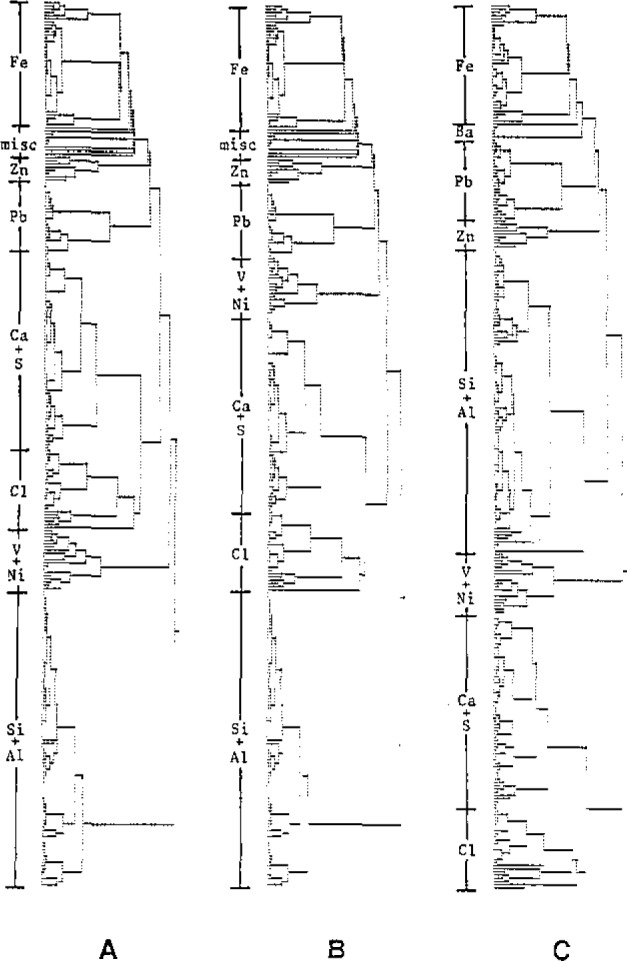
Dendrograms obtained by applying hierarchical cluster analysis to the same data set using different interpretation methods: (a) expert system, (b) manual interpretation, (c) conventional program.

As an illustration, the fine fraction of a data set of 1000 particles is considered below. After performing the qualitative interpretation of the data set in three different ways, the composition of each particle was calculated using a standardless ZAF correction procedure [4]. The resulting three data matrices were subsequently used as input to hierarchical cluster analysis (using Ward’s errors sum strategy) to extract information on the different types of particles present in the sample. The resulting dendrograms are shown in figure 3. Although at first glance dendrogram C differs greatly from dendrograms A and B, roughly the same groups of particles can be distinguished. When the soil dust (Si,Al) or gypsum (Ca,S) groups are compared among the three dendrograms, approximately the same mean composition is obtained. However, when the particles containing heavy metals are considered, significant differences appear:

- In all dendrograms the Pb group consists of two subgroups. In A and B, the first subgroup mainly contains Pb (70%) and Br (24%), the second almost pure Pb (93%). In dendrogram C, however, both groups also contain S, with mean compositions of 60%Pb, 20%Br, 8%S and 82%Pb, 11%S respectively. Clearly, method C could not distinguish between S-Kα and Pb-Mα while method A could.
- Similarly, in the Zn containing particles, Na is found in all cases by method C since Na-K overlaps with Zn-Lα while no Na is found by either the manual or expert system interpretation methods.
- In the V,Ni group, two subgroups also appear, one contains soil dust elements and the other does not (methods A and B). In dendrogram C however, because the interpretation program found Cr in some of these particles, two other groups (containing Cr and not containing Cr) were formed. In this case, the interpretation errors not only yield incorrect particle compositions but have the more important effect of influencing the way particles are clustered together by introducing non-existent correlations.
- In dendrograms A and B, a number of particles of miscellaneous composition is present which do not belong to any of the larger groups. Among them is a group of five Ti particles and one Ba particle. Also pure As and Se particles are present and were identified by both the expert system and the human interpreter. In dendrogram C, however, the As particle belongs to the Pb group while a cluster of six Ba particles is present. Although the significance of these few particles in the entire data set is very small, this shows that the expert system is also capable of handling exotic particles correctly while the conventional program is clearly not.

A significant drawback in the use of the expert system is the considerable amount of computer memory and time it requires. While the conventional program takes about 5 min CPU-time to interpret a data set of 1000 particles, the expert system takes approximately 30 min, depending on the number of peaks in each spectrum.

## Conclusion

In this work, an expert system, implemented in the language OPS5, for the automated interpretation of large EPMA data sets is discussed. The interpretation results were evaluated by comparing them with those obtained from a manual interpretation method and from a conventional interpretation program using a windowing technique. This comparison shows that the results produced by the expert system are in good accordance with the results obtained by manual interpretation since the expert system is able to deal with the frequently occurring case of spectral overlap and retains only physically realistic identification possibilities.

## Figures and Tables

**Table 1a:** Peak table

Peak Number	Intensity (counts)	x-ray energy (keV)
1	167	1.244
2	212	1.479
3	1147	1.742
4	379	3.682
5	1724	4.511
6	301	4.928
7	289	5.894
8	12873	6.392
9	1666	7.043

**Table 1b:** Identification matrix obtained after data reduction of a spectrum collected from a Mg, Al, Si, Al, Ti, Mn and Fe containing particle

Possible Identifications						
K*α*	K*β*	L*α*	L*β*1	*Lβ*2	L*γ*	M*α*
Mg	**	As	**	**	**	**
Al	**	Br	**	**	**	**
Si	* *	**	**	**	**	**
Ca	**	**	Sn	**	**	**
Ti	**	Ba	**	**	**	**
V	Ti	**	**	**	**	**
Mn	Cr	**	**	**	**	**
Fe	**	**	**	**	**	**
**	Fe	**	**	**	**	**

**Table 2a:** Example of one of the expert systems meta-rules

Rule	Go back to “CLEAN” phase after “INTENS”
If	the current interpretation phase is “INTENS“ one or more “INTENS”-rules has been used
Then	modify the interpretation phase to “CLEAN” set the number of used “CLEAN”-rules to zero.
**OPS5-form:**	
(p intens::control:back-to-clean	
→	{<PHASE> (phase ^^^name intens ^^^intenscount > 0)} (modify <PHASE> ^^^name clean ^^^cleancount 0))

**Table 2b:** Example of one of the rules from the CLEAN module, which removes K*β*-entries for a given element if no K*α*-entry is found in the data base

Rule	Remove isolated K*β*-entries from the database
**If**	the current interpretation session is “CLEAN” a peak is found corresponding to the K*β*-line of an element
	no peak is found which corresponds to the K*α*-line of this element
**Then**	remove the K*β*-entry from the data base.
**OPS5-form:**	
(p clean::k:kb-no-ka	
	{<PHASE> (phase ^^^name clean ^^^cleancount <n>)}
	{<KB-PEAK> (peak ^^^kb (<el> <> **})} —(peak ^^^ka <el>)
→	(modify <KB-PEAK> ^^^kb **)
	(modify <PHASE> ^^^cleancount (compute <n> + 1)))
